# Mitochondrial VDAC1-based peptides: Attacking oncogenic properties in glioblastoma

**DOI:** 10.18632/oncotarget.15455

**Published:** 2017-02-17

**Authors:** Anna Shteinfer-Kuzmine, Tasleem Arif, Yakov Krelin, Shambhoo Sharan Tripathi, Avijit Paul, Varda Shoshan-Barmatz

**Affiliations:** ^1^ Department of Life Sciences and the National Institute for Biotechnology in the Negev, Ben-Gurion University of the Negev, Beer-Sheva 84105, Israel

**Keywords:** apoptosis, glioblastoma, mitochondria, peptides, VDAC1

## Abstract

Glioblastoma multiforme (GBM), a primary brain malignancy characterized by high morbidity, invasiveness, proliferation, relapse and mortality, is resistant to chemo- and radiotherapies and lacks effective treatment. GBM tumors undergo metabolic reprograming and develop anti-apoptotic defenses. We targeted GBM with a peptide derived from the mitochondrial protein voltage-dependent anion channel 1 (VDAC1), a key component of cell energy, metabolism and apoptosis regulation. VDAC1-based cell-penetrating peptides perturbed cell energy and metabolic homeostasis and induced apoptosis in several GBM and GBM-derived stem cell lines. We found that the peptides simultaneously attacked several oncogenic properties of human U-87MG cells introduced into sub-cutaneous xenograft mouse model, inhibiting tumor growth, invasion, and cellular metabolism, stemness and inducing apoptosis. Peptide-treated tumors showed decreased expression of all tested metabolism-related enzymes and transporters, and elevated levels of apoptotic proteins, such as p53, cytochrome *c* and caspases. Retro-Tf-D-LP4, containing the human transferrin receptor (TfR)-recognition sequence, crossed the blood-brain barrier (BBB) via the TfR that is highly expressed in the BBB to strongly inhibit tumor growth in an intracranial xenograft mouse model. In summary, the VDAC1-based peptides tested here offer a potentially affordable and innovative new conceptual therapeutic paradigm that might overcome GBM stemness and invasiveness and reduce relapse rates.

## INTRODUCTION

Glioblastoma multiforme (GBM) is the most common and aggressive malignant adult primary brain tumor, as it is highly invasive and proliferative, and resistant to standard therapeutic strategies [1]. GBM is a heterogeneous cancer with distinct phenotypic properties, including transient quiescence, self-renewal and adaptation to hypoxia. The intra-tumoral heterogeneity underlies the failure of conventional and targeted therapies to achieve long-term remissions [2, 3]. Indeed, 88% of GBM patients succumb to the disease within 14-36 months [4].

The majority of cancer types, including GBM, undergo alterations, including those leading to protection from apoptosis, and metabolic reprograming, considered a downstream consequence of tumor development and oncogene activation [5]. Evasion of apoptosis is a prominent hallmark of cancer, with alterations in cancer cells resulting in impaired apoptotic signaling, which facilitates tumor development and metastasis. Indeed, resistance to apoptosis is a major obstacle for most cancer therapeutics, with tumor cells employing various molecular mechanisms to suppress apoptosis and acquire resistance to apoptotic agents [5–7]. These include expression of anti-apoptotic proteins, such as Bcl-2, or down-regulation or mutation of pro-apoptotic proteins, such as Bax. Indeed, several Bcl-2 family proteins have been implicated in the pathogenesis of gliomas [8, 9].

Cancer cells undergo metabolic reprograming that includes alterations in cellular metabolism, bioenergetics and increased aerobic glycolysis (the “Warburg effect”). These changes provide tumors with precursors for the biosynthesis of nucleic acids, amino acids, phospholipids, fatty acids, cholesterol and porphyrins, molecules that are recruited for fueling survival in unfavorable micro-environments [5, 10]. This phenotype may promote a state of apoptosis resistance [11]. Similar to other cancers, GBM cells undergo metabolic reprogramming to promote proliferation and survival [1].

In this study, we targeted the cancer hallmarks of reprogrammed metabolism and apoptosis avoidance using voltage-dependent anion channel 1 (VDAC1)-based cell-penetrating peptides. VDAC1, found in the outer mitochondrial membrane (OMM), has been identified as a dynamic regulator of global mitochondrial function, under normal physiological conditions as well as during disease states [12–14]. VDAC1, a multi-functional protein, is found at the crossroads of metabolic and survival pathways and is now accepted as a master-gatekeeper that regulates the flux of metabolites and ions between mitochondria and the cytoplasm [14] and also participates in apoptotic cell death [12–14].

VDAC1 is over-expressed in many cancer types [13], including GBM [15], where it contributes to the metabolic phenotype of cancer cells via the transport of various metabolites, nucleotides, Ca^2+^ and other ions across the OMM. VDAC1 binding of hexokinase (HK-I and HK-II) regulates the glycolytic flux [16] and also appears to protect tumor cells from cell death [14, 17–19].

Along with regulating cell energy status, VDAC1 is involved in the process of mitochondria-mediated apoptosis by mediating the release of apoptotic proteins [12–14]. VDAC1 also regulates apoptosis via binding of apoptosis-regulating proteins, such as HK, Bcl-2 and Bcl-xL, some of which are also highly expressed in many cancers. We have identified VDAC1 domains and amino acid residues important for such interactions and have designed VDAC1-based peptides targeting these interactions, thereby inhibiting the anti-apoptotic effects of these proteins [17, 18, 20–22]. We have previously reported that cell-penetrating VDAC1-based peptides induce cell death in cancerous but not in non-cancerous cells [23]. Moreover, these peptides effectively induced cell death in a variety of cancer cell lines, regardless of their cancer origin or the mutations carried, including mutated or absent p53. Furthermore, the peptides showed a triple-mode of action, namely impairing cell energy homeostasis, prevention of the protective effect of anti-apoptotic proteins and induction of massive apoptosis [13, 23]. Therefore, VDAC1-based peptides can be considered as pan-drugs, representing potential therapeutic candidates for treating many cancers, including GBM.

Here, we report that VDAC1-based cell-penetrating peptides induced apoptotic cell death in several brain cancer-derived cell lines and perturbed cell energy homeostasis. In sub-cutaneous and intracranial-orthotopic GBM xenograft mouse models, VDAC1-based peptides inhibited tumor development by inducing massive apoptotic cell death involving enhanced expression of pro-apoptotic proteins, eliminating cancer stem cells (CSCs) and reversing the metabolic reprograming of the cancer cells.

## RESULTS

**Figure 1:**
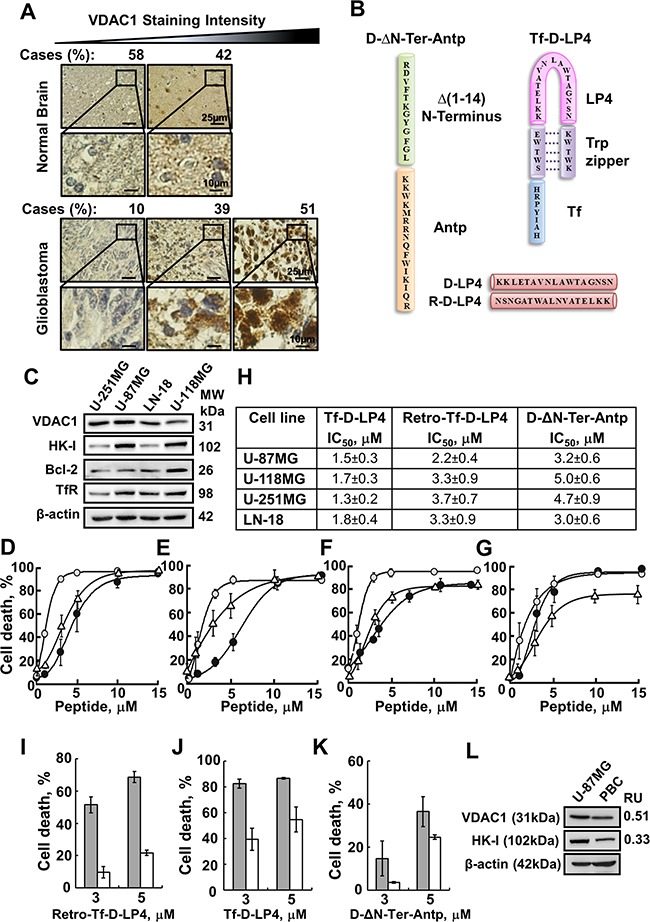
VDAC1-based peptides induce dramatic cell death of several brain tumor-derived cell lines but to lesser extent in primary brain cells **A**. IHC staining of VDAC1 of human normal brain (n=13) or glioblastoma (n=41) in tissue array slides (Biomax), as described in Materials and methods available online in Supplemental information. Percentages of sections stained at the intensity indicated are shown. **B**. Schematic illustration of D-ΔN-Ter-Antp (left) and Tf-D-LP4 (right) peptides. The VDAC1-derived sequences Δ(1-14)N-terminus and LP4 are in green and pink, respectively. The cell-penetrating peptides Antp and Tf are in orange and blue, respectively. The loop shape of LP4 stabilized by a tryptophan zipper is in purple. The amino acids of Δ(1-14)N-terminus, LP4 and Antp are in the D configuration. The sequences of D-LP4 and Retro-D-LP4 are presented below. **C**. Immunoblot analysis of VDAC1, HK-I, Bcl-2 and Transferrin receptor (TfR) expression in brain tumor-derived cell lines using specific antibodies, as described in Materials and methods available online in Supplemental information. **D-G**. D-ΔN-Ter-Antp, Tf-D-LP4 and Retro-Tf-D-LP4 peptides effectively induce cell death of human brain tumor-derived cell lines. Cells (U-251MG (D), U-118MG (E), U-87MG (F), LN-18 (G)) were incubated with the Tf-D-LP4 (○), D-ΔN-Ter-Antp (·) or Retro-Tf-D-LP4 (Δ) peptide in the appropriate serum-free growth medium for 6 h at 37°C. Cells were harvested, washed twice with PBS and cell death was analyzed by propidium iodide (PI) staining and flow cytometry. **H**. IC_50_ values (μM) for pepdide inducing cell death as derived from D-G. The results shown correspond to means ± SD (n=3). **I-K**. Retro-Tf-D-LP4, Tf-D-LP4 and D-ΔN-Ter-Antp peptides induced cell death to a lesser extent in primary brain cells (PBCs), as compared to U-87MG cells. U-87MG cells (grey bars) and primary brain cells (white bars) were incubated with the indicated concentrations of Retro-Tf-D-LP4 (I), Tf-D-LP4 (J) or D-ΔN-Ter-Antp (K) peptide in the appropriate serum-free growth medium for 6 h at 37°C. Cells were harvested, washed twice with PBS and cell death was analyzed by PI staining and flow cytometry. **L**. Immunoblot analysis of VDAC1 and HK-I expression levels in U-87MG cells and PBCs using specific antibodies was carried out as described in the Materials and Methods section available online in Supplemental Information. The levels of VDAC1 and HK-I in PBCs are presented relative to levels in U-87MG cells and relative to β-actin levels.

In this study, we assessed the effect of synthetic cell-penetrating VDAC1-based peptides in inducing cell death in GBM and characterized their mode of action both *in vitro* and *in vivo*. VDAC1 is over-expressed in a variety cancer types [13], including GBM (Figure 1A), suggesting its important metabolic function in the growth of cancer cells. VDAC1 over-expression results in apoptotic cell death [14, 24], yet GBM cells survive. This suggests that the resistance of GBM to apoptosis is most likely conferred by over-expression of VDAC1-interacting anti-apoptotic proteins, such as HK and Bcl-2 (Figure 1C) [9, 25]. We thus tested the possibility of interfering with the apoptosis-suppressive capacity of such proteins, using VDAC1-mimetic peptides to induce apoptosis in a broad range of cell lines derived from various cancers, regardless of the carried mutations.

### The mode of action of VDAC1-based peptides *in vitro* – apoptosis induction

In our previous study [23], we designed and tested over 40 versions of VDAC1-based cell-penetrating peptides to identify the most stable short apoptosis-inducing peptides. Of these multiple versions, we selected the VDAC1-based peptides D-Δ(1-14)N-Ter-Antp (D-ΔN-Ter-Antp) and Tf-D-LP4, representing two different structural parts of VDAC1 and most active in cell death induction, for use in this study (Figure 1B). D-ΔN-Ter-Antp is composed of Antp (Penetrating), a 16 residue-long sequence from the *Drosophila* antennapedia-homeodomain, fused to a VDAC1-N-terminal sequence, both containing amino acids in the D-configuration, to make them more resistant to proteolytic degradation. Tf-D-LP4 is a cell-penetrating peptide comprised of a VDAC1-derived sequence, defined as LP4, fused to a human transferrin receptor (hTfR)-recognition sequence, HAIYPRH (Tf) [26], with only the amino acids of the VDAC1-derived sequence being in D-configuration. hTfR is highly-expressed in many cancers [26], thus allowing targeting of the peptide to cancer cells. GBM patient-derived cell lines, such as U-87MG (mutated PTEN), CRL-2610 (LN-18) (mutated PTEN and p53), U-251MG (mutated PTEN and p53) and U-118MG (mutated PTEN and p53), showed high expression of TfR (Figure 1C).

To address the change of orientation due to the D-configuration of the amino acids, we also designed a retro-inverso analogue of the Tf-D-LP4 peptide (Figure 1B). Retro-inverso peptides are peptides in which the sequence, including D-amino acids, is reversed, such that the α-center chirality of the amino acid subunits is also inverted. The reverse sequence helps maintain side chain topology, similar to that of the original L-amino acid peptide.

Incubation of human GBM cell lines (U-87MG, U-118MG, U-251MG and LN-18) with the VDAC1-based peptides resulted in marked cell death, as monitored using propidium iodide (PI) staining and flow cytometry analysis (Figure 1D–1G). The data were fitted to calculate the peptide concentration required for half-maximal cell death activity (IC_50_) values obtained for D-ΔN-Ter-Antp, Tf-D-LP4 and Retro-Tf-D-LP4 are presented (Figure 1H). Similar results were obtained with the human neuroblastoma SH-SY5Y, mouse neuroblastoma Neuro-2a and mouse glioblastoma GL-261MG cell lines (Supplementary Figure 1A-1D).

The D-ΔN-Ter-Antp, Tf-D-LP4 and Retro-Tf-D-LP4 peptides were found to induce cell death to a lesser extent in mouse primary brain cells (PBCs), as compared to U-87MG cells (Figure 1I-1K). Immunoblot analysis of VDAC1 and HK-I expression showed that their levels in PBCs were about 2-fold lower than in U-87MG cells (Figure 1L).

**Figure 2:**
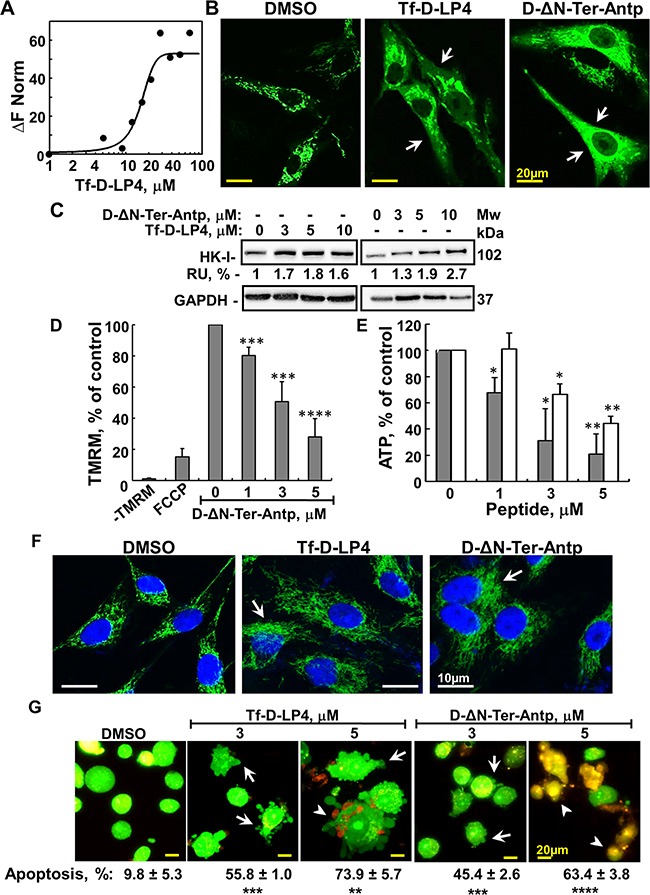
Mode of action of VDAC1-based peptides – interacting with- and detaching HK, releasing Cyto *c*, decreasing ATP level and inducing apoptosis **A**. Purified HK-II was fluorescently labeled using the NanoTemper protein-labeling Kit BLUE. HK-II (100 nM) was incubated with Tf-D-LP4 (1- 100 μM for 20 min). Then, 3-5 μl of the samples were loaded into MST-grade glass capillaries and thermophoresis was measured using a Monolith-NT115 apparatus, as described in Materials and methods available online in Supplemental information. A Kd of 16 μM was determined for HK-II. **B**. Tf-D-LP4 and D-ΔN-Ter-Antp induce HK-I-GFP detachment. U-87MG cells (1×10^5^/ml) were transfected with pEGFP-HK-I and after 24 h, incubated with Tf-D-LP4 or with D-ΔN-Ter-Antp (7μM) for 3 h in serum-free medium. The final DMSO concentration in control and peptide-treated cells was 0.07%. Fixed cells were visualized using confocal microscopy (Olympus 1×81), as described in Materials and Methods available online in Supplemental information. Arrows indicate cells with diffusion of HK-I-GFP. **C**. For HK detachment, U-87MG cells were incubated with or without the indicated concentration of Tf-D-LP4 and D-ΔN-Ter-Antp for 3 h, treated with digitonin (0.02%) and HK in the cytosolic fraction was analyzed by immunoblotting, as described in Materials and Methods available online in Supplemental information. Anti-GAPDH antibodies were used to verify the cytosolic extracts. The levels of HK in the supernatants were quantified and are presented as relative units. **D**. D-ΔN-Ter-Antp induces mitochondrial inner membrane depolarization analyzed as described in Materials and Methods available online in Supplemental information. U-87MG cells were incubated for 3 h with the indicated concentrations of D-ΔN-Ter-Antp or with FCCP (1 h, 25 μM). Cells then were analyzed for Δψ using TMRM. Δψ is presented as percentage of control. Results = mean ± SE (n=3) (*** p≤0.001, ****p ≤0.0001). **E**. D-ΔN-Ter-Antp (grey bars) and Tf-D-LP4 (white bars) reduce cellular ATP levels assayed as described in Materials and Methods available online in Supplemental information. Cellular ATP levels are presented as percentage of control. Results = mean ± SE (n=2 or 3) (* p≤0.05, ** p≤0.01). **F**. Tf-D-LP4 and D-ΔN-Ter-Antp induce Cyto *c* release. U-87MG cells were incubated with Tf-D-LP4 or with D-ΔN-Ter-Antp (10 μM) for 3 h in serum-free medium. Release of Cyto *c* from the mitochondria was analyzed by immunostaining using anti-Cyto *c* antibodies and confocal microscopy (Olympus 1×81) as described in Materials and Methods available online in Supplemental information. Arrows indicate cells showing diffusion of Cyto *c*. **G**. Tf-D-LP4 and D-ΔN-Ter-Antp induce apoptotic cell death. U-87MG cells were treated with the indicated concentrations of Tf-D-LP4 and D-ΔN-Ter-Antp for 3 h and then stained with acridine orange and ethidium bromide (100μg/ml). Arrows and arrowheads indicate cells with membrane blebbing (early apoptotic state) and late apoptotic states, respectively. Quantification of apoptosis in control and peptide's treated cells are presented bellow the images. Results = mean ± SE (n=3) (** p≤0.01, *** p≤0.001, **** p≤0.0001).

Next, to characterize the mode of action of the peptides, their effects on cell energy production and apoptosis induction were tested. As various studies have demonstrated that the glycolytic enzyme HK is aberrantly expressed in GBM, where it is an important mediator of aerobic glycolysis, providing a proliferative and cell survival advantage [27] and knowing that VDAC1-based peptides interact with HK [16–20], the interaction of Tf-D-LP4 with HK was analyzed (Figure 2A). Using MST, an approach that enables evaluating VDAC1-based peptide interaction with HK and extraction of a binding affinity coefficient (Kd) [28], a Kd value of 16.6 μM for HK-II was revealed (Figure 2A).

Peptide-induced HK-I detachment from VDAC1 was analyzed by expressing HK-I-GFP and visualizing its cellular distribution (Figure 2B and Supplementary Figure 2A). HK-I-GFP fluorescence was punctuated, as expected for mitochondrial distribution, but became diffuse upon exposure to Tf-D-LP4 or D-ΔN-Ter-Antp peptide, reflecting HK-I-GFP detachment from the mitochondria (Figure 2B and Supplementary Figure 2A). HK detachment by the peptide was also demonstrated by following its presence in the cytosolic fraction of peptide-treated cells (Figure 2C).

The effects of the D-ΔN-Ter-Antp and Tf-D-LP4 peptides on mitochondrial membrane potential (ΔΨm) and cellular ATP levels were analyzed. Treatment of U-87MG cells with the D-ΔN-Ter-Antp but not with Tf-D-LP4 peptide (data not shown) resulted in reduced tetramethylrhodamine, methyl ester (TMRM) fluorescence, reflecting a decrease in ΔΨm (Figure 2D). Similarly, as expected from the decrease in ΔΨm, cellular ATP levels were dramatically decreased by about 80% in the D-ΔN-Ter-Antp-treated cells, and by about 50% in cells treated with Tf-D-LP4 (Figure 2E). These results show that peptide treatment dramatically decreased cell energy production. The results suggest that the decrease in energy production resulted from an effect on glycolysis mediated by HK detachment from VDAC1, as well as on oxidative phosphorylation.

The activity of the D-ΔN-Ter-Antp and Tf-D-LP4 peptides in inducing cytochrome c (Cyto *c*) release was analyzed in U-87MG cells by immunofluorescence (IF) using anti-Cyto *c* antibodies. As we found that upon peptide inducing cell death, Cyto *c* was degraded, we analyzed short incubation time with the peptides and follow Cyto *c* release using IF. Representative confocal images of control cells showed that the fluorescence is punctuated, suggesting mitochondrial distribution (Figure 2F). Under the conditions used, the peptide induced partial Cyto *c* release reflected in diffused fluorescence in the cytoplasm (Figure 2F).

Apoptotic cell death, as induced by Tf-D-LP4 and D-ΔN-Ter-Antp peptides and analyzed by cell staining with acridine orange/ethidium bromide, showed cells with membrane blebbing, a hallmark of apoptosis, and ethidium bromide staining, reflecting a late apoptotic stage (Figure 2G and Supplementary Figure 2B). Quantitation of these results showed that in the conditions used, about 63% to 74% of the cells underwent apoptosis upon incubation with 5 μM of the peptide (Figure 2G).

Apoptosis induction in D-ΔN-Ter-Antp peptide-treated U-87MG cells was also analyzed by electron microscopy (Supplementary Figure 2C). Representative micrographs showed that in both control and peptide-treated cells, the plasma membrane was visibly intact. Only peptide-treated cells showed morphological changes that included condensation of nuclei, DNA fragmentation, and possible apoptotic body formation, all typically associated with apoptosis.

### VDAC1-based peptides inhibit tumor growth *in vivo*

**Figure 3:**
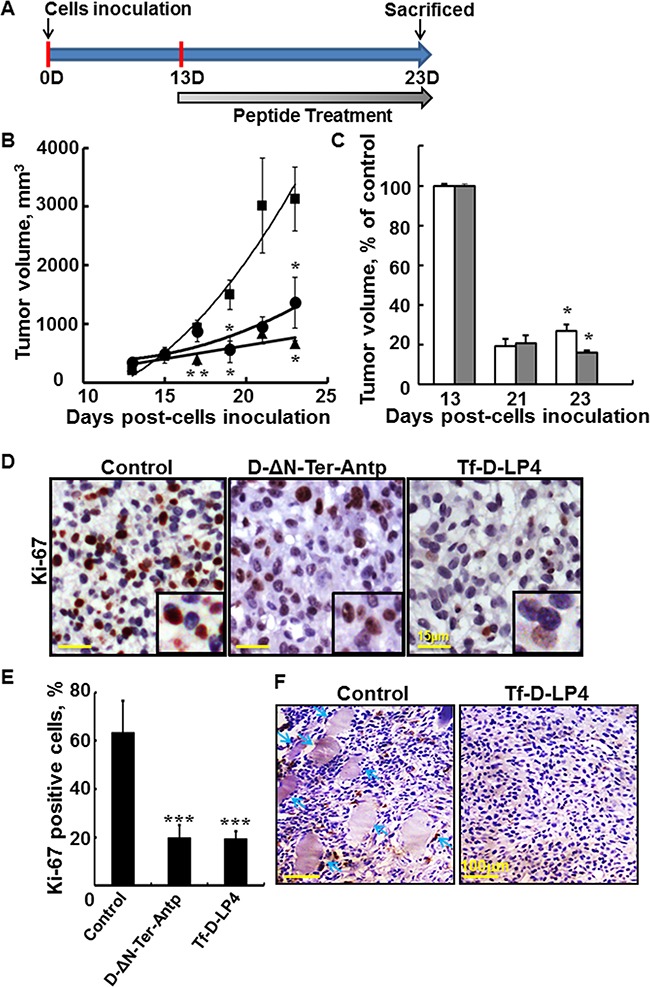
VDAC1-based peptides inhibit tumor growth, cell proliferation, and invasion in a glioblastoma xenograft mice model **A**. Graphical representation of the xenograft experiment protocol used. U-87MG cells (3×10^6^/mouse) were inoculated into male nude mice as described in Materials and Methods available online in Supplemental information. **B, C**. Tf-D-LP4 and D-ΔN-Ter-Antp inhibit tumor growth. **B**. On day 13, when tumor volume was 100-130 mm^3^, mice were sub-divided into 3 matched groups (5 mice per group), and injected every two days with DMSO (■, control, 0.26%), D-ΔN-Ter-Antp, (·, 20 μM) or Tf-D-LP4 (▲, 20 μM). The calculated average tumor volumes are presented as means ± SE (n=5) (* p≤0.05, ** p≤0.01). **C**. The volume of peptide-treated tumors is presented as % of control for each indicated time. Results = mean ± SE (n=5) (* p≤0.05, ** p≤0.001). **D**. Tf-D-LP4 and D-ΔN-Ter-Antp peptides decrease cell proliferation. Representative sections from PBS/DMSO, Tf-D-LP4 and D-ΔN-Ter-Antp peptide-TTs were immunostained for Ki-67, hematoxylin-stained and visualized by light microscopy. **E**. Quantitative analysis of Ki-67 positive cells. Results = mean ± SE (n=5) (*** p<0.001). **F**. H&E staining of control and peptide-treated U-87MG tumors showing representative sections with arrows pointing to muscle, indicative of tumor invasion.

Next, we tested the effects of the D-ΔN-Ter-Antp and Tf-D-LP4 peptides *in vivo*, using a GBM xenograft mouse model (Figure 3A). Nude mice were injected sub-cutaneously (s.c) with U-87MG cells. Following tumor formation (100-130 mm^3^), the mice were split into three tumor volume-matched groups. Each group was treated intra-tumorally with PBS containing DMSO at a final concentration of 0.26% (PBS/DMSO, control), or with D-ΔN-Ter-Antp or Tf-D-LP4 peptides (20 μM) and tumor growth was followed. In untreated tumors, volume grew exponentially and increased over 30-fold in 2 weeks, while the volume of peptide-treated tumors (TTs) only slightly increased (Figure 3B). At the end point, the average tumor volume in peptide-injected tumors was lower by about 75% and 85% for D-ΔN-Ter-Antp and Tf-D-LP4, respectively, relative to the PBS/DMSO-injected tumors (Figure 3C). Finally, the mice were sacrificed and the tumors were excised and subjected to hematoxylin-eosin (H&E) or immunohystochemistry (IHC) staining.

As the peptides also induced a decrease in cell energy (Figure 2D, 2E), we analyzed the effects of the peptides on cell proliferation capacity by IHC of tumor sections using anti-cell proliferation factor Ki-67 antibodies (Figure 3D, 3E). Representative paraffin-embedded sections from PBS/DMSO, D-ΔN-Ter-Antp- and Tf-D-LP4-TTs revealed strong staining of the nuclei in the PBS/DMSO-TTs, staining that was pronouncedly lower in the peptide-treated residual tumors. Quantitative analysis indicated that Ki-67-positive cell numbers were lower by about 45% in Tf-D-LP4-TTs and in D-ΔN-Ter-Antp-TTs (Figure 3E).

VDAC1-based peptide treatment also eliminated tumor invasion, as reflected by the fact that sub-cutaneous tumors treated with PBS/DMSO reached the muscle, while no such invasion was observed in the peptide-TTs (Figure 3F).

### VDAC1-based peptides induce apoptosis and over-expresion of pro-apoptotic proteins *in vivo*

**Figure 4:**
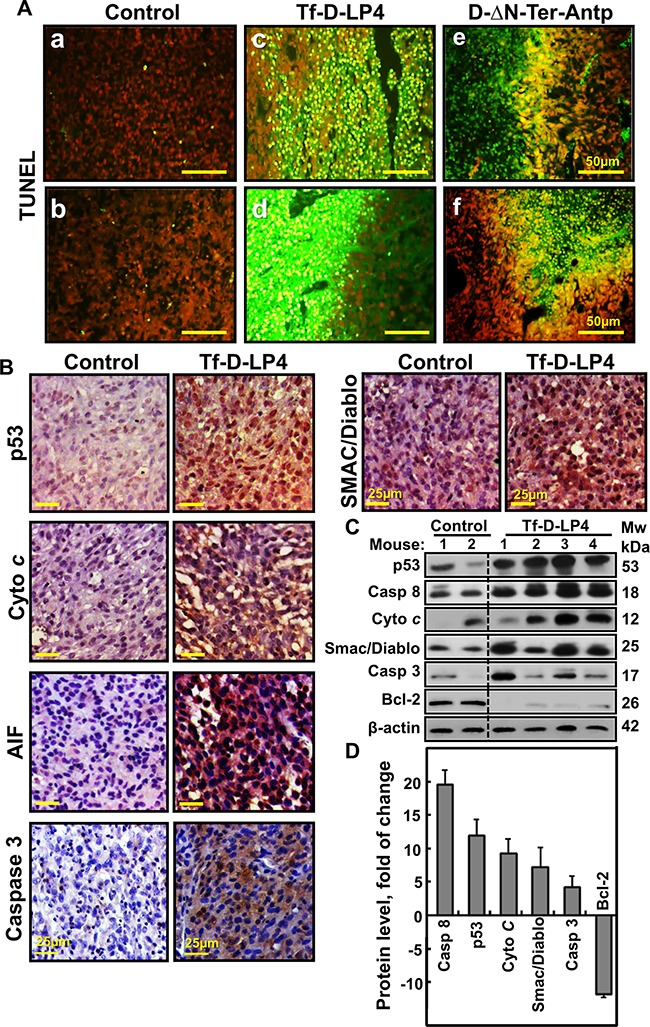
Tumor treatment with VDAC1-based peptide induced apoptosis and modified the expression of apoptosis-related proteins **A**. TUNEL staining on paraffin-embedded sections cut from (**a, b**) PBS/DMSO-, and (**c, d**) Tf-D-LP4 or (**e, f**) D-ΔN-Ter-Antp peptide-TTs from dissected mouse xenografts. Red color indicates PI nuclear staining and green color TUNEL staining. **B**. Representative sections from PBS/DMSO- and peptide-TTs IHC stained for p53, Cyto *c*, AIF, Caspase 3 and Smac/Diablo and then hematoxylin-stained and visualized by microscopy. **C**. Tissue lysates obtained from controls and several Tf-D-LP4-TTs were immunoblotted for p53, Caspase 8, Cyto *c*, Smac/Diablo, Caspase 3 and Bcl-2. **D**. Quantitative analysis of immunoblots is shown (n=2-4).

The marked decrease in tumor size in the peptide-treated U-87MG-derived xenografts can be attributed to both inhibition of cell proliferation (Figure 3D, 3E) and to peptide-induced cell death. The presence of apoptotic cells *in situ* was analyzed by TUNEL staining of tissue sections cut from PBS/DMSO- and peptide-TTs (Figure 4A). While a few TUNEL-positive cells were apparent in PBS/DMSO-TTs, the majority of the cells were TUNEL-positive in the Tf-D-LP4- and D-ΔN-Ter-Antp-TTs, with staining co-localizing with PI nuclear staining (Figure 4A). While the majority of section areas were TUNEL-positive, some areas were not, most probably due to a lower extent of delivery of the peptide. H&E staining also revealed apoptotic cell death in sections from peptide-TTs but not in PBS/DMSO-TTs (Supplementary Figure 3). The results thus indicate that, as with cells in culture (Figures 1D-1G, 2G and Supplementary Figures 1A-1C and 2B, 2C), intra-tumoral peptides also induced apoptotic cell death. As similar results were obtained with both Tf-D-LP4- and D-ΔN-Ter-Antp-TTs, the following analyses present results only for Tf-D-LP4-TTs.

Given that one of the hallmarks of cancer is avoidance of apoptosis [5, 10], and that the mode of action of the tested peptides involves apoptosis induction (Figures 2G, 4A and Supplementary Figure 2B, 2C), the expression levels of apoptosis-related proteins in PBS/DMSO- and Tf-D-LP4-TTs were analyzed (Figure 4B–4D). Due to its central role in activating U-87MG expressing p53, yet p53 expression cellular responses to stress, particularly DNA repair, p53 is frequently absent or mutated in cancerous cells [29]. p53 expression levels were highly elevated (over 12-fold) in Tf-D-LP4-TTs (Figure 4B–4D). Caspase 8, mediating the apoptosis extrinsic pathway, was over-expressed by about 20-fold, and the expression of caspase 3 was also increased (>5-fold) in the peptide-TTs (Figure 4B–4D). Cyto *c*, a key player in mitochondria-mediated apoptosis, was also highly expressed (over 10-fold) in the peptide-TTs (Figure 4B–4D). Expression levels of the pro-apoptotic protein SMAC/DIABLO was much higher in peptide-TTs, while the level of the anti-apoptotic proteins Bcl-2 was almost undetectable (Figure 4B-4D).

### VDAC1-based peptides alter the expression levels of metabolic enzymes and transporters *in vivo*

**Figure 5:**
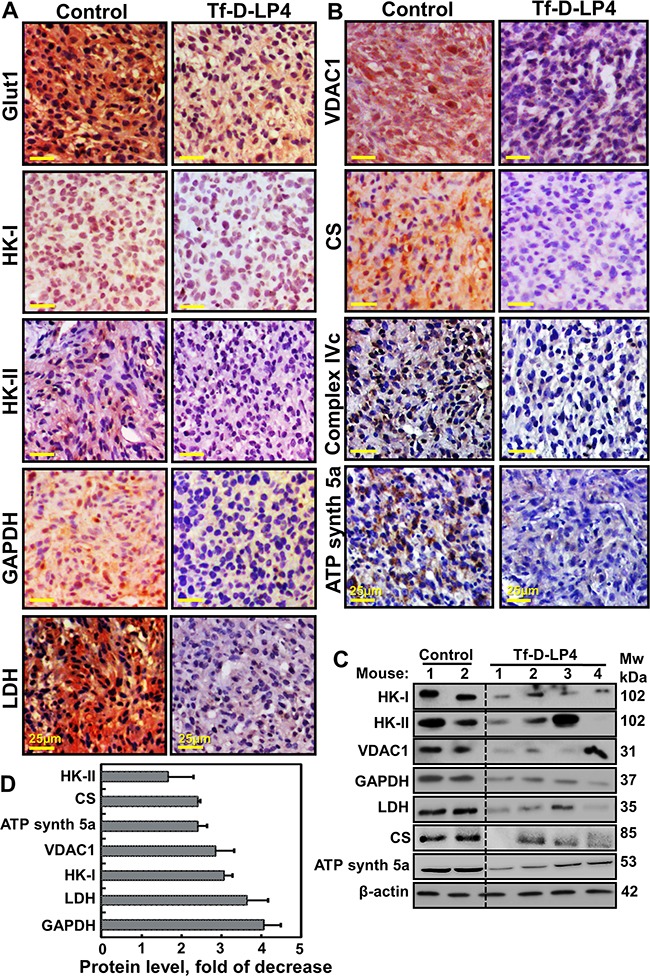
VDAC1-based peptide-treated tumors show marked changes in the expression of energy- and metabolism-related enzymes Dissected tumors were subjected to immunohistochemistry as described in Experimental Procedures. **A, B**. Representative sections from PBS/DMSO- and Tf-D-LP4-TTs were IHC stained for Glut-1, HK-I, HK-II, GAPDH, LDH, VDAC1, CS, Complex IVc and ATP synthase 5a are presented. Sections were also hematoxylin-stained and visualized by microscopy. **C**. Tissue lysates obtained from controls and several Tf-D-LP4-TTs were immunoblotted for HK-I, HK-II, VDAC1, GAPDH, LDH, CS, and ATP synthase 5a. **D**. Quantitative analysis of immunoblots is shown (n=2-4).

As cancer cells undergo metabolic changes while maintaining high levels of glycolytic activity essential for cancer cell survival and growth [10], the metabolic features of control and Tf-D-LP4-TTs were analyzed using IHC and immunoblotting (Figure 5). U-87MG xenografts showed high expression levels of a glucose transporter (Glut-1) and the glycolytic enzymes HK-I, HK-II, glyceraldehyde dehydrogenase (GAPDH) and lactate dehydrogenase (LDH) (Figure 5A, 5C, 5D and Supplementary Figure 4). Strikingly, the levels of these enzymes were dramatically lower in the Tf-D-LP4-TTs. The expression levels of the Kreb's cycle enzyme citrate synthase (CS), the mitochondrial electron transport protein complex IVc, and ATP synthase subunit 5a were also markedly lower in peptide-TTs (Figure 5B-5D).

VDAC1, a key protein in cancer cell energy and metabolism homeostasis, is highly expressed in GBM (Figure 1A, 1C) [15]. In U-87MG cells, strong VDAC1 staining was obtained in control, PBS/DMSO-TTs sections, while only very weak staining was seen in Tf-D-LP4-TTs (Figure 5B–5D) and ΔD-N-Ter-Antp-TTs (Supplementary Figure 4). Immunoblotting of representative proteins and their quantitative analysis (Figure 5B-5D) further demonstrated the marked changes in expression of metabolic proteins as induced by tumor treatment with the Tf-D-LP4 peptide. These findings point to a reversal of the metabolic reprograming of the cancer cells in response to VDAC1-based peptide action.

### The VDAC1-based peptides eliminated cancer stem cells in tumors and induced cell death in cultured cancer stem cells

**Figure 6:**
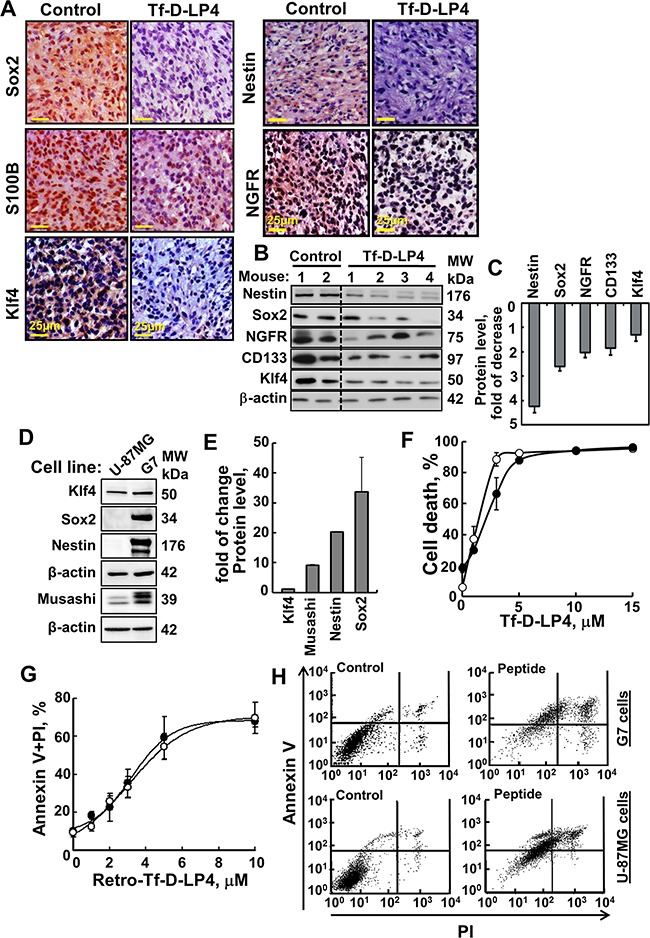
VDAC1-based peptide treatment modifies the expression of GSCs and effectively induces cell death of stem cells cell line G7 **A**. Representative sections from PBS/DMSO- and Tf-D-LP4-TTs subjected to IHC staining of Sox2, S100B, Klf4, Nestin and NGFR as described in Experimental Procedures are presented. Sections were also hematoxylin-stained and visualized by microscopy. **B**. Tissue lysates obtained from controls and several TF-D-LP4-TTs were immunoblotted for Nestin, Sox2, NGFR, CD133, and Klf4. **C**. Quantitative analysis of immunoblots is shown (n=2-4). **D**. Immunoblot analysis of, Klf4, Sox2, Nestin and Musashi expression in U-87MG and G7 cell lines, using specific antibodies. **E**. Quantitative analysis of immunoblots. **F**. Tf-D-LP4 peptide effectively induces cell death of G7 stem cells. G7 (·) and U-87MG cells (○) (2×10^5^/ml) were incubated for 6 h with DMSO (0.15%) or Tf-D-LP4 peptide in serum-free growth medium at 37°C and then analyzed for cell death using PI staining and flow cytometry. **G-H**. Retro-Tf-D-LP4 peptide induces apoptosis of U-87MG and the stem cell G7 cell line. **G**. G7 (·) and U-87MG cells (○) (2×10^5^) were incubated for 3 h with the DMSO (0.15%) or with the indicated concentration of Retro-Tf-D-LP4 peptide in serum-free growth medium at 37°C and then subjected to FITC-Annexin V/PI staining and flow cytometry analysis. **H**. Representative FACS analysis of DMSO- (left) and 5 μM of Retro-Tf-D-LP4- (right) treated G7 or U-87MG cells.

The effects of peptide treatment on tumor cell energy status and on apoptosis induction led us to speculate that the peptide may also affect other tumorigenesis-associated processes. Therefore, we next compared the expression levels of selected proteins involved in stemness in PBS/DMSO- and Tf-D-LP4-TTs using IHC and immunoblotting (Figure 6).

Accumulating evidence suggests the existence of a small reservoir of glioma cells that share properties with neural stem cells and which are capable of driving tumorigenesis. These cells, termed GBM stem cells (GSCs), are capable of extensive proliferation, self-renewal and multi-lineage differentiation [3, 30, 31]. Therefore, we analyzed the expression levels of GSC markers, including Sox2, CD133, Klf4, S100B, Nestin and NGFR, in PBS/DMSO- and Tf-D-LP4-TTs. Expression levels of these proteins, as analyzed by both IHC and immunoblotting, were markedly lower in the Tf-D-LP4-TTs, in some cases undetectable (Figure 6A–6C).

To further demonstrate the effect of the peptide on GSCs, we used the cancer stem cell G7 cell line derived from a GBM patient [32] and tested its sensitivity to peptide-induced cell death in comparison to that of U-87MG cells (Figure 6F–6H). The GSCs-specific markers, Sox2, Musashi, and Nestin, were highly expressed in G7 cells, as compared to U-87MG cells, containing about 1% GSCs, thus confirming the stemness of the cells (Figure 6D, 6E). Klf4 is expressed in both cell lines with its level in G7 being slightly higher. The peptide Tf-D-LP4 induced cell death in G7 cells with a similar efficacy as in the U-87MG cell line (Figure 6F). The concentrations for inducing half-maximal cell death (IC_50_) were 1.5±0.3μM and 2.5±0.3μM for U-87MG and G7 cells, respectively. Similar results were obtained with Retro-Tf-D-LP4 and when apoptosis was assayed using annexinV/PI staining and FACS analysis (Figure 6G–6H).

The results thus clearly indicate that GSCs are sensitive to the VDAC1-based peptide.

### Retro-Tf-D-LP4 peptide, free or encapsulated in PLGA nano-particles, is able to cross the blood-brain barrier *in vivo*, where it is effective in inducing apoptosis-inhibited intracranial tumor growth

**Figure 7:**
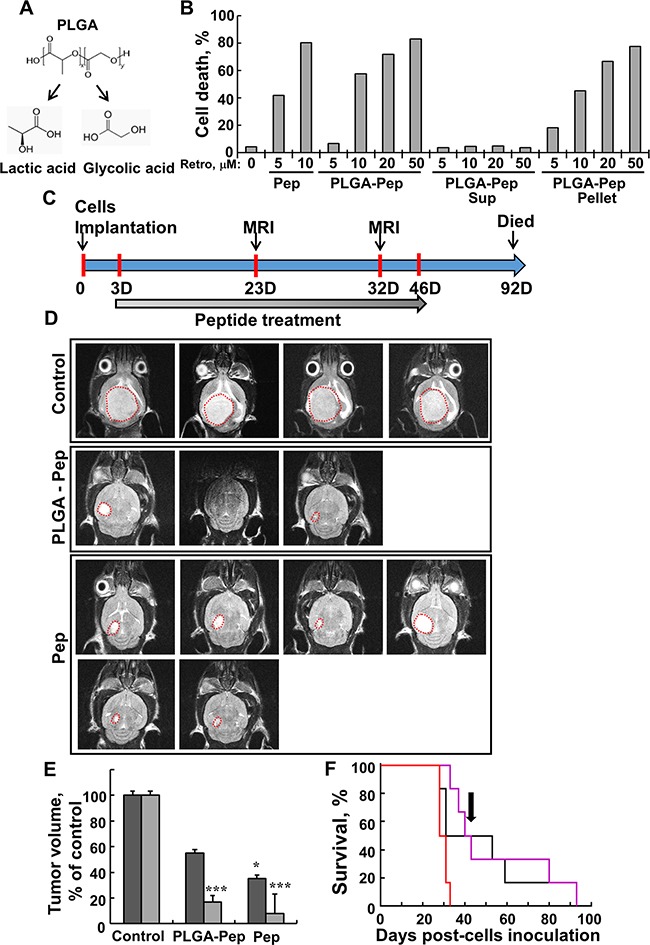
Free- and PLGA-encapsulated peptide induces cell death in U-87MG cells and reduces tumor volume in a U-87MG intracranial glioblastoma mouse model **A**. Structure of PLGA and its degradation products, glycolic acid and lactic acid. **B**. U-87MG cells (2×10^5^) were incubated for 12 h with the indicated concentrations of DMSO, Retro-Tf-D-LP4 peptide (Pep), or Retro-Tf-D-LP4 encapsulated in PLGA nanoparticles (PLGA-Pep). Samples of Retro-Tf-D-LP4 encapsulated in PLGA nanoparticles were centrifuged (15000g, 5 min) to separate the supernatant (Sup), containing free peptide, and the pellet, containing encapsulated Retro-Tf-D-LP4 peptide. Cell death was analyzed by PI staining and FACS analysis. **C**. Graphical representation of the orthotopic animal experiment. **D**. MRI imaging of brains 29 days post-intravenal treatment start with DMSO (1.05%), Retro-Tf-D-LP4 (10 mg/kg) encapsulated in PLGA nanoparticles or Tf-D-Retro-LP4 peptide (10 mg/Kg), as described in Materials and Methods available online in Supplemental information. **E**. Calculated tumor volumes after 20 (dark grey bars) and 29 (light grey bars) days post-treatment start. Results = mean ± SE (n = 6) (* p<0.05, *** p<0.001). **F**. Kaplan-Meier survival curves showing statistically significant differences in survival curves between the PBS/DMSO and Retro-Tf-D-LP4 treated mice. Cumulative Kaplan-Meier survival curves of control mice (red line), Retro-Tf-D-LP4 peptide (10mg/kg) encapsulated in PGLA nanoparticles (black line, ** p=0.006) or Retro-Tf-D-LP4 peptide (10 mg/kg, pink line, *** p=0.0003). The arrow indicates the end of the peptide treatment (day 46).

One main obstacle to treating GBM is delivering a therapeutic drug to the affected area across the blood-brain barrier (BBB). The BBB is highly restrictive and selective, allowing passage of only very small molecules (<600 Da) or peptides that pass by diffusion or via specific transporters. Drugs encapsulated in nanoparticles [33] or conjugated to a sequence that is recognized and imported by cell receptors [34] are able to cross the BBB. Here, we used Retro-Tf-D-LP4 peptide, assuming it would cross the BBB via the TfR, highly expressed in BBB [35, 36], and using nanoparticles made of poly lattice-co-glycolide (PLGA) (Figure 7A), allowing controlled release of the cargo.

To demonstrate that the peptide was encapsulated within the PLGA nanoparticles and active, the particles were centrifuged and the cell death-inducing activity of the resulting supernatant and re-suspended nanoparticles was analyzed (Figure 7B). The results clearly indicated that the peptide was encapsulated in the PLGA nano-particles and induced cell death in U-87MG cells.

Brain orthotopic tumor models currently offer the best way to study the characteristics of a brain tumor in the context of a live animal, particularly at sites with unique physiological and architectural qualities, such as the brain. These models allow for assessment of features such as metabolism, drug delivery across the BBB, and toxicity. Thus, to better mimic the clinical situation of GBM, we used intracranial-orthotopic xenografts [37] to examine peptide effectiveness in inhibiting tumor growth (Figure 7C–7E). U-87MG cells were engrafted into nude mice brains, and the mice were treated intravenously (i.v.) with PBS/DMSO, free- or PLGA-encapsulated-Retro-Tf-D-LP4, and 20 and 29 days later, tumor growth was monitored by MRI (Figure 7D, 7E and Supplementary Figure 5). Decreases of 60% and 90% in orthotopic xenograft tumor volume were obtained when mice were treated intravenously with free Retro-Tf-D-LP4 peptide (10 mg/kg) 20 and 29 days after the start of treatment, respectively. Similarly, treatment PLGA-encapsulated (10 mg/kg) Retro-Tf-D-LP4 resulted in decreases of 45% and 80% in orthotopic xenograft tumor volume 20 and 29 days after the start of treatment, respectively (Figure 7E).

Finally, analysis using Kaplan-Meier survival curves revealed statistically significant differences in survival between the PBS/DMSO and free- or PLGA-encapsulated Retro-Tf-D-LP4-treated mice (Figure 7F). Peptide treatment was ended 46 days post-cell inoculation and mice survival was followed for another 46 days. Although peptide treatment was terminated, the treatment prolonged the survival of 40 and 50% of the free- or PLGA-encapsulated Retro-Tf-D-LP4-treated mice, respectively, and by over 60 days, relative to untreated mice which all died by day 32.

The results suggest that both free- and PLGA-based peptide preparations reached the brain and were effective in cell death induction in an intracranial tumor, with free Retro-Tf-D-LP4 being more effective both in reducing tumor size and in prolonging mice survival.

## DISCUSSION

The high heterogeneity of the GBM tumor, harboring multiple genetic alterations, may contribute to the failure of conventional treatments, as well as of the wide array of recently developed novel therapeutic approaches [38]. Thus, treatment of GBM remains extremely challenging and requires new molecular targets and novel therapeutic approaches that also target GSCs in the tumors in order to prevent recurrence [39].

**Figure 8:**
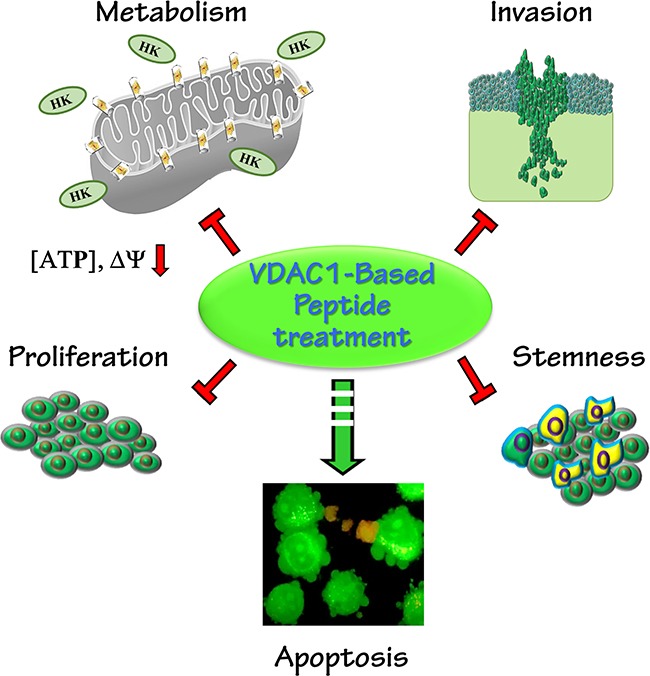
A schematic presentation of a VDAC1-based peptide inducing reversal of a tumor's oncogenic properties Tumor treatment with VDAC1-based peptides resulted in attacks on hallmarks of cancer and reversal of oncogenic properties. These included mitochondrial dysfunction, decreased energy and metabolite generation, arrested cell proliferation, induction of apoptosis, and inhibition of invasion and stemness.

Here, we used various brain-derived cell lines and sub-cutaneous and intracranial xenograft GBM mouse models to present the potential of VDAC1-based peptides as innovative and effective treatments for GBM. Our results suggest that VDAC1-based peptides have multiple effects, including perturbing cell energy homeostasis, inhibiting tumor growth, stemness and inducting apoptosis (Figure 8).

The multiple effects of these peptides, together with the apparent lack of side effects, reflected in the fact that mice which received the peptide intravenously for 4 months at higher concentration than used here showed no effect on weight, physical activity, blood glucose, or on internal organ weight or tissue morphology, make the peptide a promising anti-cancer agent.

### VDAC1-based peptides inhibit cell growth and tumor development

The cell-penetrating VDAC1-based peptides used here compete with VDAC1 for the Bcl-2-, Bcl-xL- and HK interaction sites and consequently interrupt their anti-apoptotic activities [17, 18, 20–23, 40]. VDAC1-based peptides effectively induced cell death of various neuronal and GBM-derived cell lines via perturbed cell energy status, as reflected by HK detachment, decreased Δψ and cellular ATP levels, and induced apoptosis (Figures 1, 2 and Supplementary Figures 1 and 2).

In xenografts of U-87MG cells intratumorally treated with Tf-D-LP4 or D-ΔN-Ter-Antp peptides, tumor growth was highly inhibited (~90%). Moreover, in the intracranial-orthotopic xenograft GBM mouse model [37], mice treated i.v. with free- or PLGA nanoparticle-encapsulated Retro-Tf-D-LP4 showed significantly lower tumor volume (by up to 90% for free peptide) (Figure 7D, 7E and Supplementary Figure 5). Furthermore, although peptide treatment was terminated, about 50% of peptide-treated mice survived some 60 days longer than untreated mice (Figure 7F). This suggests that the peptide most likely crosses the BBB via the TfR shown to be enriched in the BBB [35, 36], and was able to mediate antibody uptake by the brain [41]. The peptide also crossed the BBB when encapsulated in PLGA nanoparticles [42].

This dramatic effect of the peptides on tumor growth can be attributed to their action on two major cancer hallmarks, namely the cancer cell energy status and induction of apoptotic cell death.

### VDAC1-based peptides alter cancer cell energy status

The use of glucose as an energy source via glycolysis is a feature common to most solid tumors [43], including GBM cells [44] but not GSCs [45]. Alterations in glucose metabolism have been correlated with carcinogenesis, cancer progression and even response to treatment [46]. VDAC1, as a gatekeeper for the entry and exit of mitochondrial metabolites that also interacts with proteins that mediate and regulate the integration of mitochondrial functions with cellular activities, contributes to the metabolic phenotype of cancer cells. Thus, its over-expression in many cancer types [13], including GBM (Figure 1A) [15], is not surprising. Furthermore, over-expressed VDAC1 presents anchoring sites for over-expressed HK. VDAC1-bound HK not only lies at the apex of the glycolytic pathway but also protects against mitochondria-mediated cell death and decreases ROS production [14]. To attack the metabolic benefit gained by cancer cells due to VDAC1-bound HK, many compounds designed to interfere with glycolysis by targeting HK, such as by 2-deoxyglucose (2-DG) and 3-bromopyruvate (3-BP), that also targets GAPDH, and lonidamine (a derivate of indazole-3-carboxylic acid), were developed [47, 48]. However, these glycolysis inhibitors are not very potent and are toxic to certain normal tissues, including brain, retinae, and testes, as these also use glucose as their main energy source [47]. Furthermore, progenitor cells and GSCs from all GBM lines tolerate inhibition of glycolysis or oxidative phosphorylation, and only the combined inhibition of both pathways leads to a substantial depletion of intracellular ATP [45]. On the other hand, disruption of VDAC1-HK-interaction by the peptides presented here provides a strategy to interfere with cancer cell growth and activate apoptosis and can thus be considered as a promising approach for cancer therapy [13, 47].

VDAC1-based peptides interact with and detach HK from its binding site in VDAC1, yet affect overall cellular bioenergetics. This is reflected in the peptide-induced decrease in ΔΨm and cellular ATP levels (Figure 2D, 2E) [18, 23], and induction of cell death (Figures 1D-1G and Supplementary Figure 1).

The increased metabolic activity characteristic of malignant cells is reflected in the up-regulation of Glut1, HK and other glycolytic enzymes [49]. Strikingly, the expression levels of Glut1, HK-I, HK-II, GAPDH and LDH were prominently decreased in the peptide-TTs (Figure 5A, 5C, 5D and Supplementary Figure 4). Decreases in Kreb's cycle activity and oxidative phosphorylation are reflected in the decreased expression of CS, complex IVc and ATP synthase 5a subunits (Figure 5B–5D). The down-regulation of Glut1 and glycolysis enzymes, as well as of oxidative phosphorylation, suggests that in the residual peptide-TTs, the re-programed metabolism of the cancer cell was reversed.

The effects of VDAC1-based peptides presented here point to VDAC1 as a key protein in the metabolic adaptations attained during GBM development and invasion, with functional consequences that have not been previously reported for any GBM treatment. Thus, although targeting cancer metabolism is challenging due to the nature of metabolic plasticity, redundancy and adaptation, VDAC1, over-expressed in GBM and a key protein in regulating cancer cell energy and metabolism, is an emerging target.

### VDAC1-based peptides enhance expression levels of pro-apoptotic proteins *in vivo*, amplifying cell death

Anti-apoptotic mechanisms play an important role in the development and progression of various cancers and in the poor responses of tumors to therapeutic intervention [5]. Similarly, the pathways that control apoptosis are altered in GBM, leading to resistance towards apoptotic stimuli [50]. VDAC1 is involved in the process of mitochondria-mediated apoptosis, mediating the release of apoptotic proteins and interacting with anti-apoptotic proteins, such as HK, Bcl-2 and Bcl-x [13] [16–22], some of which are also highly expressed in many cancers, including GBM [9, 49]. Here, using the TUNEL assay, reflecting DNA degradation in tumor sections, we demonstrated that the peptides induced massive apoptotic cell death (Figure 4A).

The dramatic cell death shown in the TUNEL assay may reflect cells undergoing apoptosis by the multiple actions of the peptides, including antagonizing the protective effect of anti-apoptotic proteins, inducing over-expression of key proteins in apoptosis, including caspases 3, 8, p53, Cyto *c*, and SMAC/Diablo, while decreasing the expression of anti-apoptotic proteins, such as Bcl-2 (Figure 4B–4D). Finally, inducing additional non-autonomous apoptosis through a novel mechanism, termed apoptosis-induced apoptosis, is in agreement with the observed over-expression of caspase 8 (Figure 4C, 4D).

The increase in caspase 8 levels observed in peptide-TTs is in agreement with the finding that caspase 8 is absent or at low levels in many resected glioma samples [51]. This implies that many patients with glioma may not benefit from death ligand-based treatments, unless caspase-8 protein expression can be elevated, such as by VDAC1-based peptides, as demonstrated here.

### VDAC1-based peptides eliminate glioma stem cells *in vivo*

GBM contains a sub-population of highly tumorigenic self-renewing GSCs from which recurrent GBM is thought to derive [3, 30, 31]. As GSCs are relatively quiescent, they are resistant to conventional chemo- and radiotherapies [52, 53]. Thus, GSCs are potentially an attractive therapeutic target. VDAC1-based peptide tumor treatment eliminated GSCs, as reflected in the marked decrease in the expression levels of all tested stem cell markers, namely Sox2, Nestin, CD133, Klf4, S100B and NGFR (Figure 6). Moreover, the GBM patient-derived stable G7 glioma stem-cell line, presenting divergent gene expression signatures and reflecting distinct cancer stem cell phenotypes [32], is as sensitive to the VDAC1-based peptide as are U-87MG cells (Figure 6F–6H). These findings are most important, as they reflect the ability of the peptides to prevent tumor regrowth, stemness, invasiveness, and might prevent relapse associated with GSCs.

Finally, the multiple effects of the peptide on tumor development involving alterations in the expression of proteins associated with metabolism and apoptosis may be mediated via the proposed link between metabolism and epigenetics [54, 55], a link that has been explored in tumorigenesis [56]. As cancer cell metabolism in the tumor was highly modified by the peptide treatment, we suggest that the alterations in gene expression seen are epigenetically driven.

To conclude, the role of tumorigenic metabolic rewiring in supporting cancer proliferation is well established, as are strategies developed by cancer cells for evading apoptosis, allowing them to overcome apoptotic cell death and display chemotherapy resistance. VDAC1 not only regulates metabolism but is also an apoptotic checkpoint in stress and pathological conditions. Considering the poor prognosis of GBM patients, findings presented here point to VDAC1-based peptides as a potential new treatment for GBM and glioma. Due to their effects on cell energy and metabolism status and apoptosis induction, including in stem cells, such peptides could replace several types of drugs, acting as chemotherapy drugs inducing apoptosis or inhibiting proliferation, cell metabolism or eliminating cancer stem cells, thereby might preventing tumor invasion and relapse.

## MATERIALS AND METHODS

### Materials

Carbonyl cyanide-p-trifluoromethoxyphenylhy- drazone (FCCP), leupeptine, phenylmethylsulfonyl fluoride (PMSF), propidium iodide (PI), tetramethylrhodamine methyl ester (TMRM), 4′,6-diamidino-2-phenylindole (DAPI), trypan blue, glucose, acutase, laminin, beta-mercaptoethanol, dimethyl sulfoxide (DMSO), cytochalasin B, and poly-(D, L-lactic-co-glycolide) were purchased from Sigma (St. Louis, MO). Dithiothretol (DTT) was purchased from Thermo Fisher Sientific (Waltham, MA USA). The cell transfection agent, JetPRIME, was from PolyPlus (Illkirch, France). Annexin-V was obtained from Alexis Biochemicals (Lausen, Switzerland). Dulbecco's modified Eagle's medium (DMEM), BSA 7.5%, MEM non-essential amino acids (NEAA) and B27 and N2 supplements were purchased from Gibco (Grand Island, NY). DMEM/HAMS-F12 and the supplements fetal calf serum (FCS) and penicillin-streptomycin were purchased from Biological Industries (Beit-Haemek, Israel). Human EGF, human FGF and digitonin were purchased from Millipore (Billerica, MA). Primary antibodies used in immunoblotting and immunohistochemistry (IHC), as well as their dilutions, are listed in Table S2. Horseradish peroxidase (HRP)-conjugated anti-mouse and anti-rabbit antibodies and TUNEL and CellTiter-Glo Luminescent Cell Viability assay kits were obtained from Promega (Madison, WI). 3,3-diaminobenzidine (DAB) was obtained from (ImmPact-DAB, Burlingame, CA).

### Peptides

The following peptides were used in this study, with the cell-penetrating sequence in bold and the amino acids added to form a loop-shaped tryptophan zipper underlined:

Tf-D-LP4, HAIYPRHSWTWE-199-KKLETAVNLAWTAGNSN-216-KWTWK, Retro-Tf-D-LP4, KWTWK-216-NSNGATWALNVATELKK-199-EWTWSHRPYIAH (34 residues with D configuration 199-216) and D-ΔN-Ter-Antp, 15-RDVFTKGYGFGL-26-

RQIKIWFQNRRMKWKK (28 residues). These and other peptides used in this study were synthesized by GL Biochem (Shanghai, China) to >95% purity. The peptides were dissolved in DMSO and concentrations were determined using absorbance at 280 nm and the specific molar excitation coefficient.

### Cell culture

U-87MG, U-251MG, U-118MG, LN-18, SH-SY5Y, GL-261MG and Neuro-2a cells were maintained at 37°C and 5% CO_2_ in the recommended culture medium and supplements. The G7 glioma-derived stem cell (GSC) cell line was grown using specific glioblastoma stem cell medium, as described previously [32]. Primary astrocyte and glia cells were prepared as described previously [57]. Newborn 2-3 day old mice (C57Bl/6) were sacrificed using CO_2_ and their brains were quickly removed and digested with 0.25% trypsin for 15 min at 37°C. The cell suspension was centrifuged through a 10% FCS solution and the obtained cells were grown in DMEM with 10% FCS, 5% CO_2_ at 37°C. Cells were used within 7 days.

### Cell treatment with VDAC1-based peptides and cell death analysis

Cells (6×10^5^ /ml) were incubated in 500 μl serum-free medium with the peptide of interest for 6 h at 37°C in the presence of 5% CO2. The cells were then trypsinized, centrifuged (1500xg, 5 min), washed with PBS and analyzed for cell death using propidium iodide (PI) staining and flow cytometer (Beckton-Dickinson, San Jose, CA) and BD CellQuest Pro software. Apoptotic cell death was also analyzed by PI and annexin V-FITC staining, and by acridine orange and ethidium bromide staining [58] and light microscopy (Olympus LX2-KSP).

### Mitochondrial membrane potential (▵Ψ) and cellular ATP levels, hexokinase detachment, cytochrome c release and microscale thermophoresis (MST)

Mitochondrial membrane potential (▵Ψ) and cellular ATP levels, hexokinase detachment, cytochrome c release and Microscale thermophoresis (MST) was assayed as described previously [18, 21–23].

### Xenograft experiments

U-87MG glioblastoma cells (3 x10^6^) were inoculated s.c. into the hind leg flanks of athymic eight-week old male nude mice (Envigo). Thirteen days post-inoculation, tumor volume was measured (100-130 mm^3^) and mice were randomized into three groups (5 animals/group), treated with PBS containing 0.26% DMSO or peptide in PBS, 0.26% DMSO/20 μM every second day. At the end of the experiments, the mice were sacrificed, tumors were excised and half of each tumor was either fixed and processed for IHC or frozen in liquid nitrogen for later WB.

For the intracranial-orthotopic xenograft mouse model, U-87MG cells (8×10^4^) were engrafted into a nude mouse brain using a stereotactic device. Forty eight hours after surgery, mice were randomized into three groups (6 animals/group) and treated every third day with DMSO (1.44%), Retro-Tf-D-LP4 (10mg/Kg) or Retro-Tf-D-LP4 (10mg/Kg) encapsulated by PLGA. Mice were subjected to MRI, sacrificed; brains were excised and processed for IHC. Tumor volume was analyzed using VivoQuant 2.10 software.

The experimental protocols were approved by the Institutional Animal Care and Use Committee.

### Immunohistochemistry and immunoblotting

Formalin-fixed, paraffin-embedded sections of control and peptide-TTs were hematoxylin-eosin stained, probed with appropriate antibodies via IHC staining or immunoblotting of cells and tissue extracts.

### TUNEL assay

Paraffin-embedded -fixed tumors sections were processed for a TUNEL assay using the DeadEnd Fluorometric TUNEL system (Promega) according to the manufacturer's instructions. Sections were deparaffinized, equilibrated in PBS, permeabilized with proteinase K, post-fixed in 4% paraformaldehyde, and incubated in TdT reaction mix for 1 h at 37 °C in the dark. Slides were then washed in 2X SSC buffer and counterstained with propidium iodide (1 μg/ml), and cover-slipped with Vectashield mounting medium. Fluorescent images of apoptotic cells (green) and cell nuclei (red) were captured using a confocal microscope (Olympus 1×81).

### Statistics

Means ± SE of results obtained from independent experiments are presented. The non-parametric Mann-Whitney U test was used to compare control and experimental groups. A difference was considered statistically significant when the *P-*value was deemed <0.05 (*), < 0.01 (**), <0.001 (***) or <0.0001 (****).

## SUPPLEMENTARY MATERIALS FIGURES AND TABLES


