# Supplementary material for: Mitochondrial VDAC1-based peptides: Attacking oncogenic properties in glioblastoma
Source: Oncotarget. 2017 Feb 17;8(19):31329–46. doi: 10.18632/oncotarget.15455 (PMC5458211; doi:10.18632/oncotarget.15455)
Supplement: Supplementary file 1 [file oncotarget-08-31329-s001.pdf]

Mitochondrial VDAC1-based peptides: Attacking oncogenic properties in glioblastoma

SUPPLEMENTARY MATERIAL

SUPPLEMENTARY EXPERIMENTAL PROCEDURES

Cell culture

Human glioblastoma U-87MG, U-118MG, CRL-2610 (LN-18), U-251MG, human neuroblastoma SH-SY5Y, mouse glioma GL-261 and mouse neuroblastoma Neuro-2a cell lines were maintained at 37°C in a 5% CO_2 atmosphere in the recommended culture medium supplemented with 10% FCS, 1 mM L-glutamine, 100 U/ml penicillin, and 100 µg/ml streptomycin. The glioma-derived stem cell (GSC) G7 cell line was grown using specific glioblastoma stem cell medium, as described previously [1].

Determination of cellular ATP levels

Cellular ATP levels were estimated using a luciferase-based assay (CellTiter-Glo, Promega). U-87MG cells (6×10^5/ml) were incubated with the indicated concentrations of D-ΔN-Ter-Antp peptide for 3 h, washed twice with PBS, transferred to 96-well white plates at densities of 5×10^4 cells/ml. ATP levels were assayed according to the manufacturer's protocol and luminescence was recorded using an Infinite M1000 plate reader (Tecan, Männedorf, Switzerland).

Mitochondrial membrane potential determination

Mitochondrial membrane potential (ΔΨ) was determined using tetramethylrhodamine methylester (TMRM) dye. U-87MG cells (6×10^5/ml) were incubated with the indicated concentrations of D-ΔN-Ter-Antp peptide for 3 h, washed twice with PBS, incubated with tetramethylrhodamine methyl ester (TMRM, 800 nM, 20 min) and washed with PBS. TMRM fluorescence was measured with an Infinite M1000 plate reader. FCCP served as a control for ΔΨ dissipation.

Hexokinase detachment

To visualize peptide-induced HK detachment in the cell, U-87MG or SH-SY5Y cells ($4\text{x}10^4$/ml) were grown on coverslips and transfected with plasmid pEGFP-HK-I. Twenty-four hours later, the post-tranfection cells were incubated for 3 h with a solution containing 0.07% DMSO or Tf-D-LP4 or D-ΔN-Ter-Antp peptide (7 µM). The cells were fixed for 15 min using 4% paraformaldehyde prepared in PBS, rinsed with PBS, permabilized with 0.3% PBST, and stained with DAPI (1:2000). Cell imaging was carried out by confocal microscopy (Olympus 1X81).

Cytochrome c release assayed by immunofluorescence (IF)

U-87MG cells were treated with Tf-D-LP4 or D-ΔN-Ter-Antp peptide (10 µM, 3h), washed, paraformaldehyde-fixed (4%, 15 min), permeabilized with 0.3% Triton in PBS (5 min), incubated with anti-Cyto *c* antibodies and then with secondary Alexa Fluor 488-conjugated anti-mouse antibodies. Samples were imaged by confocal microscopy (Olympus 1X81).

Hexokinase detachment assayed by immunoblotting analysis

Hexokinase detachment from mitochondria to the cytosol was analyzed by immunoblotting. U-87MG cells (3×10^5) were exposed to Tf-D-LP4 or D-ΔN-Ter-Antp peptide (3, 5, 10 µM for 3h), harvested, washed with PBS and gently re-suspended in ice-cold buffer (100 mM KCl, 2.5 mM $MgCl_2$, 250 mM sucrose, 20 mM HEPES/KOH, pH 7.5, 0.2 mM EDTA, 1 µg/ml leupeptin, 5 µg/ml cytochalasin B and 0.1 mM phenylmethylsulfonyl fluoride) containing 0.02% digitonin and incubated for 10 min on ice. Samples were centrifuged at 12,000xg at 4°C for 10 min to obtain supernatants (cytosolic extracts free of mitochondria) that were analyzed by SDS-PAGE and immuno-probed using anti-hexokinase antibodies, and then with secondary HRP-conjugated anti-rabbit antibodies.

Microscale thermophoresis (MST)

Purified HK-II was fluorescently labeled using a NanoTemper blue protein-labeling kit. Fluorescently-labeled HK-II (100 nM) was incubated with different concentrations of Tf-D-LP4 (1.0–100 µM) in PBS buffer. After 20 min of incubation, 3–5 µl aliquots were loaded into MST-grade glass capillaries (NanoTemper Technologies) and thermophoresis was measured with a NanoTemper Monolith-NT115 system (20% light-emitting diode, 40% IR laser power).

PLGA encapsulation of the VDAC1-based peptide Retro-Tf-D-LP4

Retro-Tf-D-LP4-loaded PLGA complexes were prepared by the solvent displacement method with some modifications, as previously reported [2 3]. Twenty milligrams of Retro Tf-D-LP4 were dissolved in 40 µl of 100% DMSO and then diluted 20-fold with sterile DDW to reach a concentration of 25 mg/ml in a final DMSO concentration of 5%. PLGA (50 mg) was dissolved in acetone (1 ml). Then, 105 µl of peptide were added to the PLGA-acetone solution. The resulting peptide-PLGA-acetone mixture was added drop-wise (0.5 ml/min) into 10 ml of aqueous solution containing 1% PVA (w/v). The mixtures were stirred continuously at room temperature until complete evaporation of the organic solvent. The nanoparticles were centrifuged at 15,000g (4ºC for 20 min) and the pellet was re-suspended in sterile DDW and washed two times. The resulting pellet was mixed with HBSS solution.

Xenograft and intracranial-orthotopic mouse models

U-87MG glioblastoma cells (3 x10^6) were inoculated s.c. into the hind leg flanks of athymic eight-week old male nude mice (Envigo). Thirteen days post-inoculation, tumor volume was measured and calculated (100-130 mm^3) and mice were randomized into three groups (5 mice/group). Each treatment substance was injected into the established s,c. tumors using PBS containing 0.26% DMSO or peptide in PBS, 0.26% DMSO/20 µM). The xenografts were injected 20 µl per tumor (2 points) every two days. Beginning on the day of inoculation, mouse weight and tumor volume were monitored twice a week for a period of 23 days using a digital caliper. At the end of the experiments, the mice were sacrificed, tumors were excised and *ex vivo* weight was determined. Half of each tumor was either fixed in 4% buffered formaldehyde, paraffin-embedded and processed for IHC or frozen in liquid nitrogen for later immunoblot analysis.

To generate an intracranial-orthotopic mouse model, U-87MG glioblastoma cells were engrafted into a nude mouse brain using a stereotactic device. The anesthetized mice were immobilized in a stereotactic-head frame (Stoelting, Wood Dale, IL). A middle incision was made on the skull and a burr hole was introduced 0.5 mm anterior to the bregma and 2.5 mm lateral to the midline using a drill (Stoelting). A 31-gauge needle loaded with 10 µL PBS was used to deliver tumor cells. The needle tip was inserted into the brain 3 mm deep, relative to the skull surface, and maintained at this depth for 2 minutes before injection of tumor cells. Under sterile conditions, a 3 µL solution containing U-87MG (8×10^4) cells was injected into the brain parenchyma over a period of 3 minutes using an UltraMicroPump III (World Precision Instruments, Sarasota, FL). After infusion, the needle was left in place for 1 minute before slow withdrawal. The burr hole was sealed using sterile bone wax, and the wound was closed with 5.0 nylon surgical suture. All surgical procedures were performed under sterile conditions. Forty-eight hours after surgery, the mice were randomized into three groups (6 animals/group) and treated every third day with DMSO (1.44%), Retro-Tf-D-LP4 (10 mg/kg) or Retro-Tf-D-LP4 (10 mg/kg) encapsulated by PLGA. Mice were subjected to MRI (at days 20 and 29), sacrificed, and brains were excised and processed for IHC. Tumor volume was analyzed using VivoQuant 2.10 software. These experimental protocols were approved by the Institutional Animal Care and Use Committee of Ben-Gurion University.

Gel electrophoresis and immunoblotting

Cells or tumor tissue were lysed using lysis buffer (50 mM Tris-HCl, pH 7.5, 150 mM NaCl, 1 mM EDTA, 1.5 mM $MgCl_2$, 10% glycerol, 1% Triton X-100, supplemented with a protease inhibitor cocktail (Calbiochem, UK). Cell lysates were then centrifuged at 600xg (10 min at 4°C) and samples (10-40 µg of protein) were subjected to SDS-PAGE and immunoblotting using various primary antibodies (sources and dilutions as detailed in supplementary Table 2), followed by the appropriate HRP-conjugated secondary antibodies (i.e., anti-mouse, anti-rabbit or anti-goat). Blots were developed using enhanced chemiluminescence (Biological Industries). Band intensities were analyzed by densitometry using Multi Gauge software (Fujifilm) and the values were normalized to the intensities of the appropriate β-actin signal that served as a loading control.

IHC of tumor tissue sections

IHC staining was performed on 5 µm-thick formalin-fixed and paraffin-embedded tumor tissue sections. The sections were deparaffinized by placing the slides at 60°C for 1 h and using xylene. Thereafter, the tissue sections were rehydrated with a graded ethanol series (100%-50%). Antigen retrieval for some proteins (ATP synthase 5a, AIF, caspase 3, citrate synthase, Cyto c, cytochrome c oxidase subunit IVc, GAPDH, Glut1, HK-II, Klf4, LDH, Nestin, NGFR, P53, SMAC/Diablo, Sox2, S100b, VDAC1) was performed in 0.01 M citrate buffer (pH 6.0). For CD31, HK-I and Ki-67, antigen retrieval was performed in 10 mM Tris-EDTA (pH 9) and 0.5 M Tris (pH 10), for 30 minutes each at 95-98°C. After washing sections in PBS containing 0.1% Triton-X100 (pH 7.4), non-specific antibody binding was reduced by incubating the sections in 10% normal goat serum for 2 h. After decanting excess serum, sections were incubated

overnight at 4°C with primary antibodies (sources and dilutions used detailed in Table S2) and washed with PBST. For IHC, endogenous peroxidase activity was blocked by incubating the sections in 3% H_2O_2 for 15 min. After washing thoroughly with PBST, the sections were incubated with the appropriate secondary antibodies for 2 h. For IHC, anti-mouse, anti-goat, or anti-rabbit secondary antibodies conjugated to HRP were used. Sections were washed five times in PBST and the peroxidase reaction was subsequently visualized by incubating with DAB. After rinsing in water, the sections were counter-stained with hematoxylin, and mounted with mounting medium. Finally, the sections were observed under a microscope (Leica DM2500) and images were collected at 20× magnification with the same light intensity and exposure time. Non-specific control experiments were carried out using the same protocols but omitting incubation with the primary antibodies. Hematoxylin-eosin (H&E) staining was performed as described previously [4].

SUPPLEMENTARY REFERENCES

1. Pollard SM, Yoshikawa K, Clarke ID, Danovi D, Stricker S, Russell R et al. Glioma stem cell lines expanded in adherent culture have tumor-specific phenotypes and are suitable for chemical and genetic screens. Cell Stem Cell 2009; 4: 568-580.
2. Andrieu V, Fessi H, Dubrasquet M, Devissaguet JP, Puisieux F, Benita S. Pharmacokinetic evaluation of indomethacin nanocapsules. Drug Des Deliv 1989; 4: 295-302.
3. Das J, Das S, Paul A, Samadder A, Bhattacharyya SS, Khuda-Bukhsh AR. Assessment of drug delivery and anticancer potentials of nanoparticles-loaded siRNA targeting STAT3 in lung cancer, *in vitro* and *in vivo*. Toxicol Lett 2014; 225: 454-466.
4. Elsam J. Histological and Histochemical Methods: Theory and Practice, 5(th) edition. Biotech Histochem 2008; 91: 145.

SUPPLEMENTARY FIGURES AND TABLES

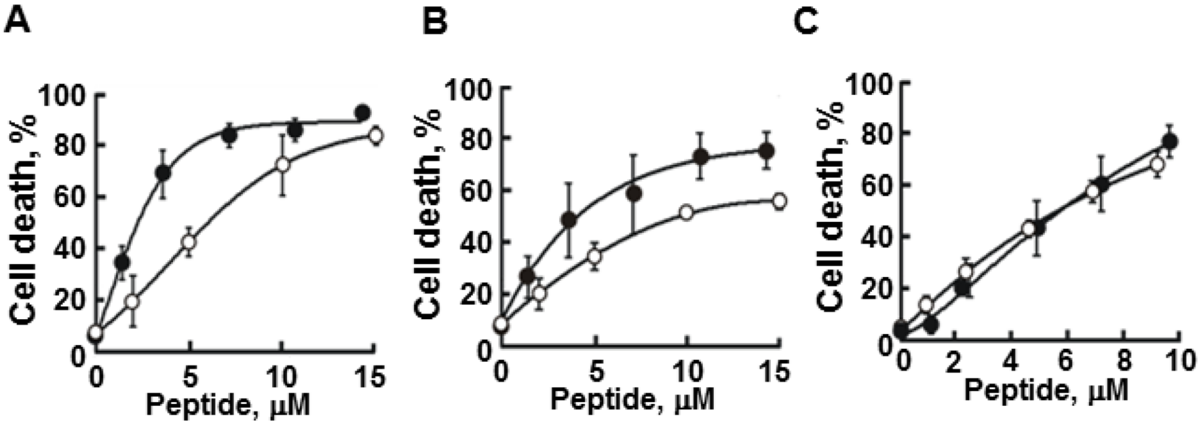

D

Cell line	Tf-D-LP4 IC_{50}, µM	D-ΔN-Ter-Antp IC_{50}, µM
**SH-SY5Y**	5.9±1.2	2.2±0.2
**GL-261MG**	3.5±0.3	4.1±0.6
**Neuro-2a**	4.5±0.5	3.8±1.4

Supplementary Figure 1: VDAC1-based peptides induce dramatic cell death of several brain tumor-derived cell lines. A-C. D-ΔN-Ter-Antp and Tf-D-LP4 peptides effectively induce cell death of brain tumor-derived cell lines. SH-SY5Y (human neuroblastoma), Neuro-2a (mouse neuroblastoma) and GL-261 (mouse glioblastoma) cells were incubated with the Tf-D-LP4 (○) or D-ΔN-Ter-Antp (●) peptide in serum-free appropriate growth medium for 6 h at 37°C. Cells were harvested, washed twice with PBS and cell death was analyzed by propidium iodide (PI) staining and flow cytometry. **D.** Summary of IC_{50} values (µM) for the peptides with the indicated cell lines (n=3).

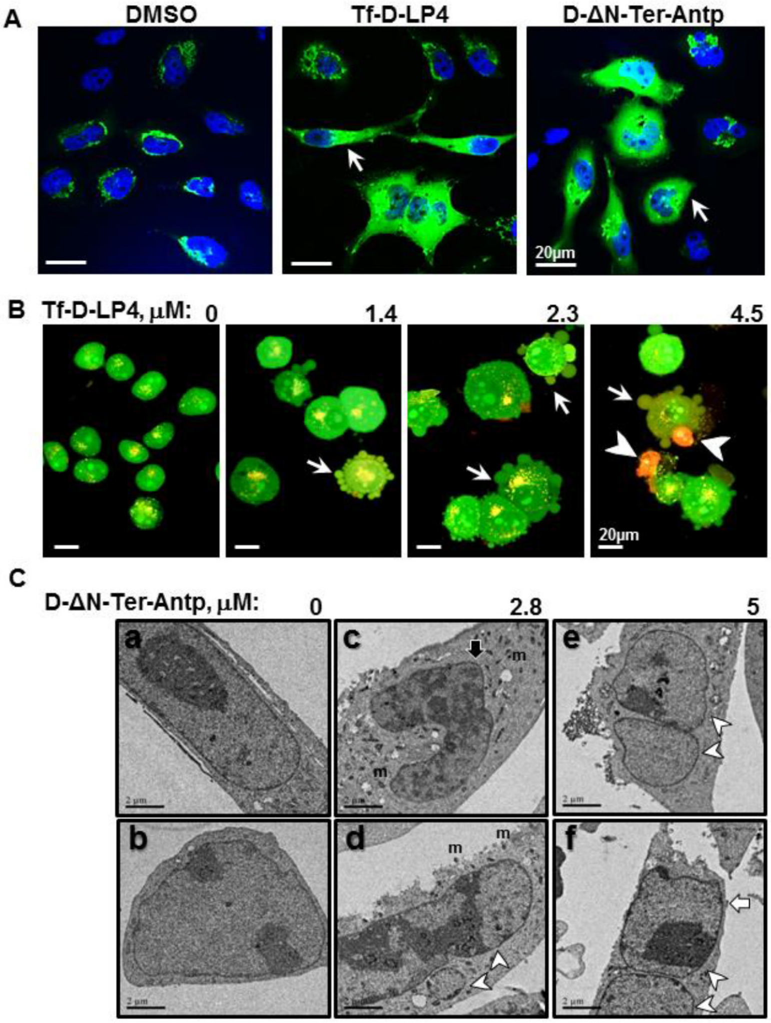

Supplementary Figure 2: Mode of action of VDAC1-based peptides. A. Tf-D-LP4 and D-ΔN-Ter-Antp induce HK-I-GFP detachment. SH-SY5Y cells (4×10^4/ml) were grown on cover slips, transfected with plasmid pEGFP-HK-I and after 24 h, were incubated with Tf-D-LP4 or D-ΔN-Ter-Antp (7 µM) for 3 h in a serum-free medium. The final DMSO concentration in control and peptide-treated cells was 0.07%. Fixed cells were stained with DAPI and visualized by confocal microscopy (Olympus 1X81). Arrows indicate cells showing diffusion of HK-I-GFP. **B-C.** Tf-D-LP4 and D-ΔN-Ter-Antp induce apoptosis. **B.** SH-SY5Y cells were treated with the indicated concentrations of Tf-D-LP4 for 3 h and then stained with acridine orange and ethidium bromide. Arrows and arrowheads indicate cells with membrane blebbing (early apoptotic state) and late apoptotic states, respectively. **C.** Morphological analysis of apoptosis by electron microscopy (EM). For transmission EM, U-87MG cells (1.6 x10^6 /ml) were incubated with the indicated concentrations of D-ΔN-Ter-Antp peptide or DMSO (0.3% final concentration) for 6 h in serum-free medium. Cells were pelleted and fixed in Karnovsky's fixative (2% paraformaldehyde, 2.5% glutaraldehyde in 0.1 M cacodylate buffer, pH 7.4) for 10 minutes at room temperature and then in fresh fixative for 3.5 days in the cold. Following buffer rinses, the cells were incubated with 1% osmium tetroxide in 0.1 M cacodylate buffer, dehydrated in an ethanol series, incubated with epoxy propane and embedded in Araldite resin. Ultrathin sections were cut using an UltracutUCT microtome (Leica, Austria), mounted on formvar-coated copper grids, doubly stained with uranyl acetate and lead citrate and viewed in a JEM-1230 transmission EM (JEOL, Japan). Digital images are collected with a Gatan model 830 ORIUS SC200 CCD camera using Gatan Digital Micrograph (DM) software. Arrow head, white arrow and black arrow indicate,nuclear fragmentation, intact plasma membrane and nuclear condensation, respectively. m indicates mitochondrion.

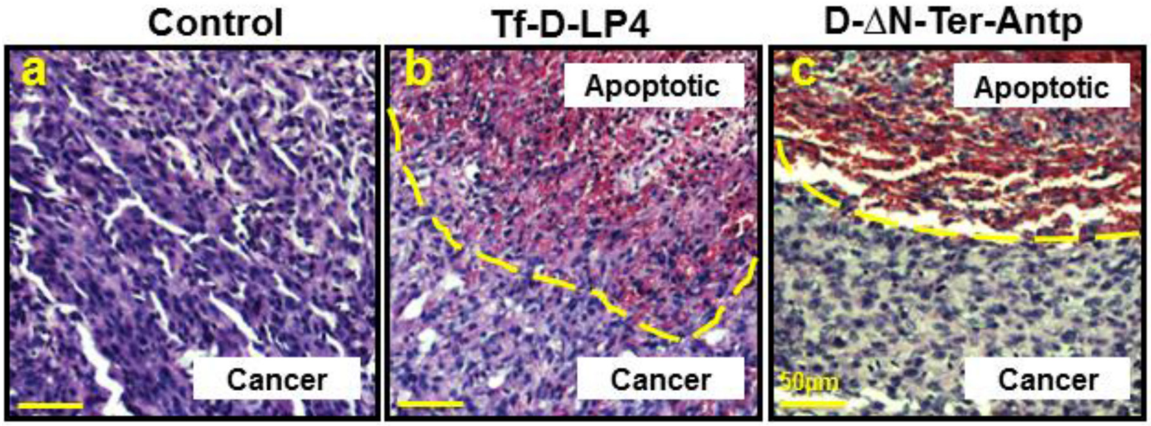

Supplementary Figure 3: Hematoxylin-eosin (H&E) staining of VDAC1-based peptide-treated tumors. Dissected tumors were subjected to H&E staining as described in Experimental Procedures. Representative sections from PBS/DMSO-, Tf-D-LP4- and D-ΔN-Ter-Antp-treated tumors were hematoxylin-eosin stained.

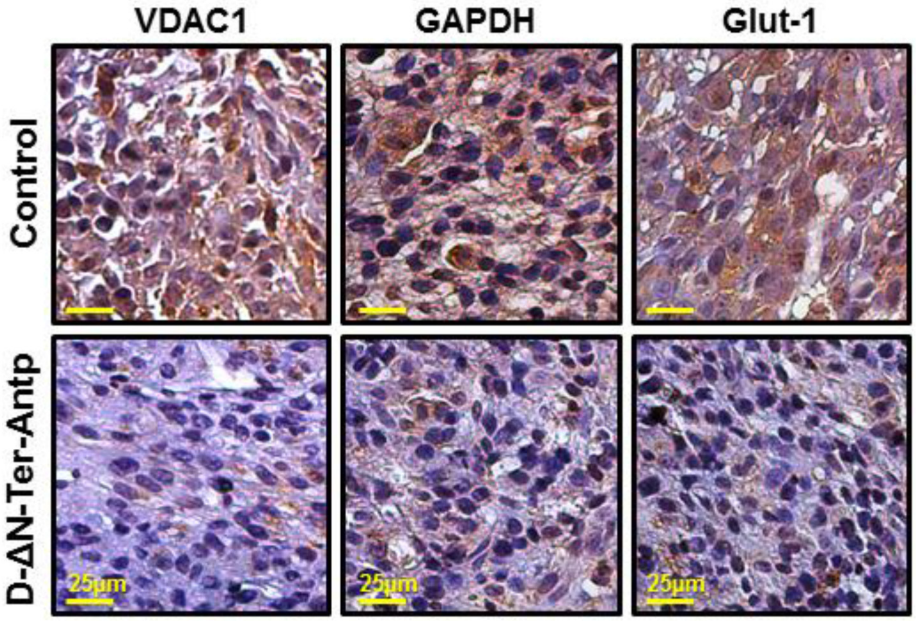

Supplementary Figure 4: VDAC1-based peptide-treated tumors show marked changes in the expression of energy and metabolic enzymes. Dissected tumors were subjected to immunohistochemistry as described in Experimental Procedures. Representative sections from PBS/DMSO- and D-ΔN-Ter-Antp-treated tumors were IHC-stained for VDAC1, GAPDH and Glut-1. Sections were also hematoxylin-stained and visualized by microscopy.

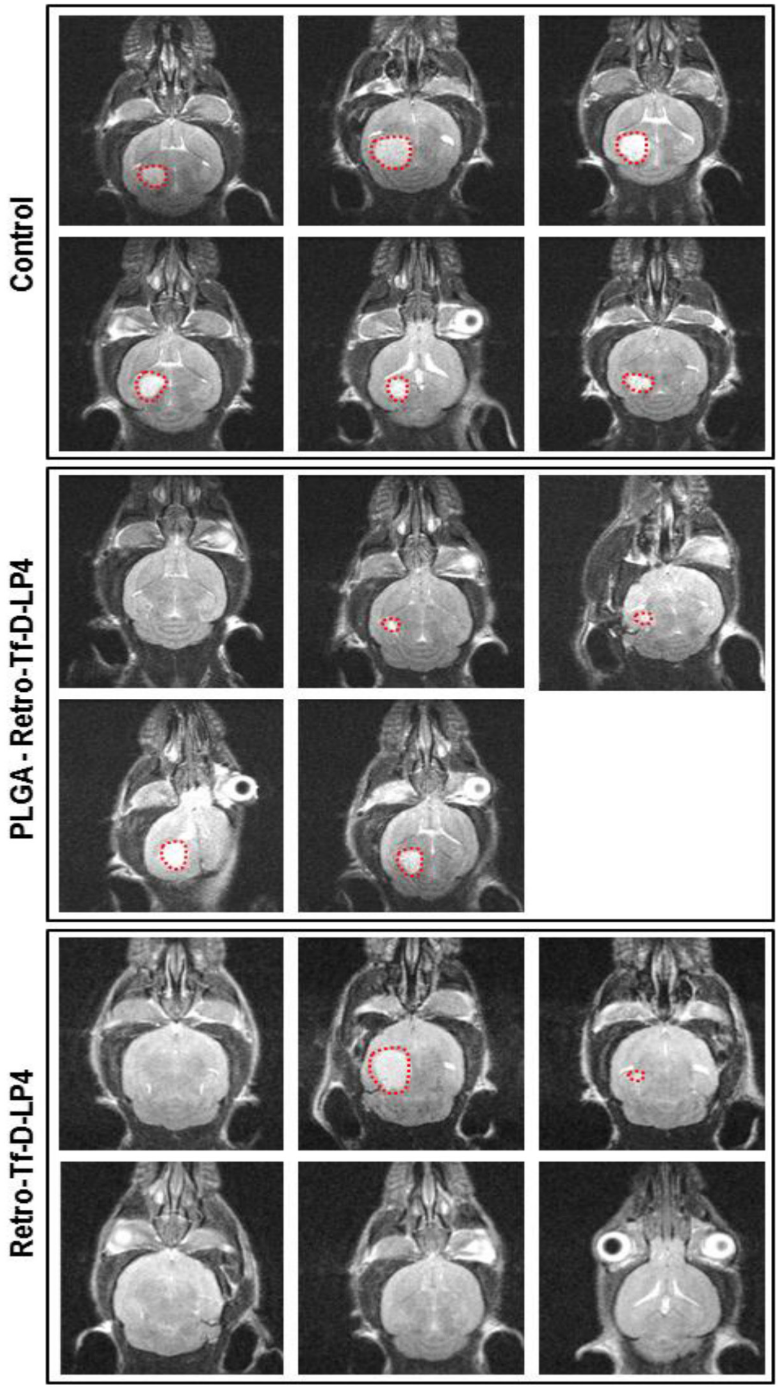

Supplementary Figure 5: Free and PLGA-encapsulated peptide reduces tumor volume in a U-87MG intracranial glioblastoma mouse mode. MRI imaging of brains 20 days post-intravenal treatment start with DMSO (1.05%), Retro-Tf-D-LP4 (10 mg/kg) encapsulated in PLGA nanoparticles or free Retro-Tf-D-LP4 peptide (10 mg/kg).

Supplementary Table 1: Amino acids sequences, MS/MS data, and analytical data for the peptides used in this study

Peptide	Sequence	No. of AA	Molecular Mass, Da	Calculated [a] molar extinction coefficient, M^{-1}	Purity %
**Tf-D-LP4**	**HAIYPRH**SWTWE-199-KKLETA VNLAWTAGNSN-216-KWTWK	34	4111.67	28990	95.94
**Retro inverse-Tf-D-LP4**	KWTWK-216-NSNGATWALNV ATELKK-199-EWTWS**HRPYIAH**	34	4111.67	28990	95.92
**D-ΔN-Ter-Antp**	15-RDVFTKGYGFGL-26-**RQIKIWFQNRRMKWKK**	28	3588.32	12490	95.79

The bold letters indicate the cell-penetrating peptide sequence while the underlined sequences represent amino acids involved in the tryptophan zipper (hairpin formation).
[a]Calculated based on amino acid composition using the following link: http://www.biomol.net/en/tools/proteinextinction.htm.

Supplementary Table 2: Antibodies used in this study, related to experimental procedures

Antibody	Source and Cat. No.	Dilution	
		IHC	WB
Mouse monoclonal anti-actin	Millipore, Billerica, MA, MAB1501	-	1:40000
Mouse monoclonal anti-ATP5a	Abcam, Cambridge, UK, ab14748	1:300	1:2000
Rabbit polyclonal anti-AIF	Abcam, Cambridge, UK, ab32516	1:200	1:1000
Mouse monoclonal anti-Bcl-2	Millipore, Billerica, MA, MAB827	-	1:2000
Mouse monoclonal anti-CD133	Miltenyi Biotec GmbH, AC133	-	1:1500
Rabbit polyclonal anti-citrate synthase	Abcam, Cambridge, UK ab96600	1:200	1:1000
Mouse monoclonal anti-cytochrome *c*	BD Bioscience, San Jose, CA, 556433		1:2000
Mouse monoclonal anti-cytochrome *c*	BD Bioscience, San Jose, CA, 556432	1:400<br>1:250 **IF**	
Rabbit monoclonal cytochrome c oxidase subunit VIc	Abcam, Cambridge, UK, ab150422	1:200	
Rabbit monoclonal anti-caspase 8	Abcam, Cambridge, UK, ab108333	-	1:1000
Mouse monoclonal anti-GAPDH	Abcam, Cambridge, UK, ab9484	1: 200	1:1000
Rabbit monoclonal anti-Glut1	Abcam, Cambridge, UK ab40084	1: 200	
Mouse monoclonal anti-HK-I	Abcam, Cambridge, UK ab105213	1:500	1:2000
Rabbit polyclonal anti-HK II	Abcam, Cambridge, UK ab3279	1:400	1:200
Rabbit monoclonal anti-Ki67	Thermo Scientific, NY RM-9106-s1	1:100	-
Rabbit polyclonal anti-Klf4	IMGENX Littleton, USA, IMG-6081-A	1:200	1:1000
Rabbit monoclonal anti-LDH	Epitomics, Cambridge, UK, 1980-1	1:300	1:1000
Mouse monoclonal anti-Musashi-1	Millipore, Billerica, MA, MABE268	-	1:2000
Rabbit polyclonal anti-Nestin	Millipore, Billerica, MA, MAB353	1:200	1:25000
Rabbit polyclonal anti -NGFR	Santa Cruz Biotechnology, Inc. Dallas, TX, sc-8317	1:200	1:1000
Mouse monoclonal anti-P53	Santa Cruz Biotechnology, Inc., Dallas, TX, sc-126	1:400	1:5000
Rabbit monoclonal anti-pro-caspase 3	Abcam, Cambridge, UK, ab32150	1:300	1:2000
Goat polyclonal anti-Sox2	Santa Cruz Biotechnology, Inc. Dallas, TX, sc-17320	1:200	1:1500
Rabbit polyclonal anti-S100B	Millipore, Billerica, MA, ABN59	1:200	-
Rabbit polyclonal anti- SMAC/Diablo	Abcam, Cambridge, UK, ab8115	1:300	1:2000
Rabbit polyclonal anti TfR	Abcam, Cambridge, UK, ab84036	-	1:2000
Rabbit monoclonal anti-VDAC1	Abcam, Cambridge, UK, ab15895	1:500	1:5000
Goat anti-Rabbit	KPL, Gaithersburg, USA, 474-1506	1:250	1:15,000
Goat anti-Mouse	Abcam, Cambridge, UK, ab97040	1:250	1:10,000
Donkey anti-Goat	Abcam, Cambridge, UK, ab97120	1:250	1:20,000
Donkey anti-Mouse (Alexa Fluor 488)	Abcam, Cambridge, UK, ab150109	1:250 **IF**	

Antibodies against the indicated protein, their catalogue number, source and the dilutions used in IHC and immunoblot (WB) experiments are presented. **IF**= immunofluoresces
